# Prevalence of ties to industry-funded organizations among patient and consumer representatives on FDA advisory committees

**DOI:** 10.1093/haschl/qxag269

**Published:** 2026-10-07

**Authors:** Genevieve P Kanter, Toni Mankowitz

**Affiliations:** Department of Health Policy and Management, Sol Price School of Public Policy, University of Southern California, VPD 512-J, 635 Downey Way, Los Angeles, CA 90089, USA; Leonard D. Schaeffer Institute for Public Policy and Government Service, University of Southern California, Los Angeles, CA 90089, USA; Leonard D. Schaeffer Institute for Public Policy and Government Service, University of Southern California, Los Angeles, CA 90089, USA

**Keywords:** FDA, advisory committee, conflict of interest, patient advocacy organization

## Abstract

**Introduction:**

FDA advisory committees are primarily composed of scientific experts, but also include a patient representative and a consumer representative providing public perspectives. Although the FDA has enacted policies to curb conflicts of interest common among its scientific members, it has paid less attention to potential conflicts arising from patient and consumer representatives who have ties to organizations financially supported by pharmaceutical companies.

**Methods:**

We analyzed the prevalence of industry funding of advocacy organizations in which patient and consumer representatives were actively involved in the year they attended FDA advisory committee meetings between 2013 and 2022.

**Results:**

Between 2013 and 2022, nearly 2 in 5 (38%) FDA advisory committee meetings included either a patient or consumer representative with active ties to a pharmaceutical-funded advocacy organization. For several FDA committees, this share was greater than 50%. More than 1 in 10 (11%) advisory committee meetings included a representative affiliated with an organization receiving funds from the sponsor of the drug under review.

**Conclusion:**

These prevalence rates show that these organizational relationships extend beyond isolated cases and signal a broader problem warranting FDA policy attention.

Key Points

- The FDA has well-established conflict-of-interest policies for its scientific advisory committee members but less developed policies for patient and consumer representatives, especially related to industry funding of these members’ advocacy organizations.
- Between 2013 and 2022, nearly 2 in 5 (38%) FDA advisory committee meetings included a patient or consumer representative with active ties to a pharmaceutical-funded advocacy organization. For some committees, this share was greater than 50%, and in some cases, the organization received financial support from the product sponsor.
- These prevalence rates show that representatives with ties to advocacy organizations funded by pharmaceutical companies or product sponsors frequently participate in FDA advisory committee meetings and signal a broader problem warranting FDA policy attention.

## Introduction

The FDA periodically convenes advisory committees consisting of external experts to advise on drug approvals and related technical issues. These committees are primarily composed of scientific experts such as clinicians and biomedical researchers, but also, by law, include a consumer representative to provide the perspective of the general public.^1,2^ Since 1991, the agency has also appointed a patient representative to provide the perspective of patients or caregivers affected by the condition under discussion.^3,4^

Although the FDA has enacted policies to curb conflicts of interest (COIs) common among its scientific members,^5^ it has paid considerably less attention to potential conflicts affecting patient and consumer representatives. The agency has largely focused on members’ direct financial ties to industry, which are more prevalent among scientific members but infrequent among patient and consumer representatives. Rather, among nonscientific members, indirect ties to industry—especially through affiliations with advocacy organizations that receive industry funding—are a particular concern. Studies have estimated that 68% of patient advocacy organizations^6^ and 83% of the largest patient organizations^7^ receive donations from drug, device, and biotechnology companies. Moreover, 12% of these patient organizations reported receiving more than half of their funding from industry.^6^ Thus, it is possible that a large number of patient and consumer representatives on FDA advisory committees have COIs related to organizational funding, and some of these conflicts may involve substantial financial dependencies, creating a risk of undue industry influence.

We analyzed the prevalence of industry funding of advocacy organizations in which patient and consumer representatives were actively involved in the year they attended FDA advisory committee meetings between 2013 and 2022.

## Data and methods

Names and organizational affiliations of patient and consumer representatives attending FDA drug advisory committee meetings were extracted from meeting rosters and transcripts, which were downloaded from the FDA website.^8^ Other organizational activities of these representatives—ie, organizational affiliations not disclosed at FDA meetings—were obtained through online searches. Organizations were included only if they were patient advocacy organizations, patient support groups, patient informational groups, or medical research foundations. Affiliations were only included if: (1) the representative held a senior leadership role with the organization, such as board member, executive director, or founder (“executive leadership”); (2) their stated position title indicated leadership responsibilities (“other leadership”); or (3) there was evidence of repeated engagement and activities (“active involvement”). We included affiliations even if the roles appeared to be unpaid but excluded affiliations that were determined to be short-term in duration (ie, less than 3 months), such as participation in annual events, awareness campaigns, training programs, and competitions. Donating to an organization was not considered to be an affiliation. Affiliations were included if they were active in the calendar year of the representative's meeting appearance.

Donors to affiliated organizations were extracted from organizational annual reports and websites and filtered to include only pharmaceutical companies. Pharmaceutical donors to advocacy organizations were included if the representative's affiliation was active in the year of the meeting and the donation was made that same year. If donors to an advocacy organization could not be identified for a given year, we assumed that the organization had no pharmaceutical company donors in that year. Sponsors of drugs under review were identified from the list of pharmaceutical donors.

We calculated the share of meetings in which at least one patient or consumer representative was actively involved with an organization that received pharmaceutical funding and, separately, sponsor funding. Shares were calculated for the full 10-year period, annually, and by committee.

Data collection began in 2023 and extended through 2024, with data cleaning and matching finalized in 2025 and data analysis completed in 2026. Methods are described in detail in Appendix A1.

## Results

Between 2013 and 2022, 98 patient representatives and 39 consumer representatives attended 234 voting meetings (Table 1, Panel A). On average, each meeting included one patient representative and one consumer representative. Patient representatives had a median of 1 advocacy organization affiliation when they attended an advisory committee meeting (mean 0.8), while consumer representatives had a median of zero affiliations (mean 0.9) (Table 1, Panel B).

**Table 1.** Summary statistics.

Panel A. Study units	*N*			
Number of meetings	234			
Number of meetings with patient representative	233			
Number of meetings with consumer representative	224			
Number of patient representatives	98^a^			
Number of consumer representatives	39^a^			
Number of committees	12			

Panel B. Distributional statistics				
	Mean (SD)	Median (IQR)	Min	Max
No. of patient representatives per meeting	1.0 (0.1)	1.0 (1.0, 1.0)	0.0	2.0
No. of consumer representatives per meeting	1.0 (0.3)	1.0 (1.0, 1.0)	0.0	2.0
Total no. of voting attendees per meeting	15.9 (4.5)	15.0 (13.0, 18.0)	7.0	30.0
No. of meetings attended by patient representative	2.4 (2.1)	2.0 (1.0, 3.0)	1.0	12.0
No. of meetings attended by consumer representative	6.1 (6.1)	5.0 (2.0, 8.0)	1.0	31.0
No. of advocacy organization affiliations of patient representative^b^	0.8 (0.8)	1.0 (0.0, 1.0)	0.0	3.0
No. of advocacy organization affiliations of consumer representative^b^	0.9 (1.1)	0.0 (0.0, 2.0)	0.0	4.0

Panel C. Individuals with organizational affiliations						
	Patient representatives			Consumer representatives		
	*N*	% all reps	% affiliated reps	*N*	% all reps	% affiliated reps
Total no. of representatives	97^a^			39		
Any affiliation^c^	57	58.8%	100.0%	19	48.7%	100.0%
Executive leadership	29	29.9%	50.9%	14	35.9%	73.7%
Other leadership	15	15.5%	26.3%	3	7.7%	15.8%
Active involvement	13	13.4%	22.8%	2	5.1%	10.5%
Disclosure status of affiliation^d^						
Disclosed	6		10.5%	7		36.8%
Undisclosed	51		89.5%	12		63.2%

Source: Authors’ analysis of FDA advisory committee meeting documents and original data collection on the organizational affiliations of patient and consumer representatives and the donors to those organizations, 2013-2022.

^a^There was one individual who participated in multiple meetings as a consumer representative and, in one meeting, as a patient representative. They are included in the counts of both patient representatives and consumer representatives in Panel A. In Panel C, they are included only in the calculations for consumer representatives, the role they served for most advisory committee meetings.

^b^We report the number of organizational affiliations attributed to the patient or consumer representative at the time of the advisory committee meeting they attended.

^c^Affiliations were considered to be “executive leadership” if the representative held a senior leadership role in the organization, such as board member, executive director, or founder; “other leadership” if their stated position title indicated leadership responsibilities; and “active involvement” if there was evidence of repeated engagement and activities. If an individual held multiple roles, we coded them in their highest role.

^d^“Disclosed” reports the number of representatives whose affiliations were always disclosed at meetings. “Undisclosed” reports the number of representatives who had at least one affiliation which went undisclosed at a meeting.

In the full sample, 58.8% of patient representatives and 48.7% of consumer representatives had an affiliation with an advocacy organization (Table 1, Panel C; Appendix A2). Of those with affiliations, a majority of patient representatives (50.9%) and consumer representatives (73.7%) had executive leadership roles such as board member, executive director, or founder in that organization.

Over the sample period, 38.0% of advisory committee meetings included either a patient representative or a consumer representative actively involved with a pharmaceutical-funded advocacy organization (Appendix A3). Patient representatives with such ties were present at 25.8% of meetings, and consumer representatives with those ties were at 17.4% of meetings (Figure 1, Panels A and B).

**Figure 1.**
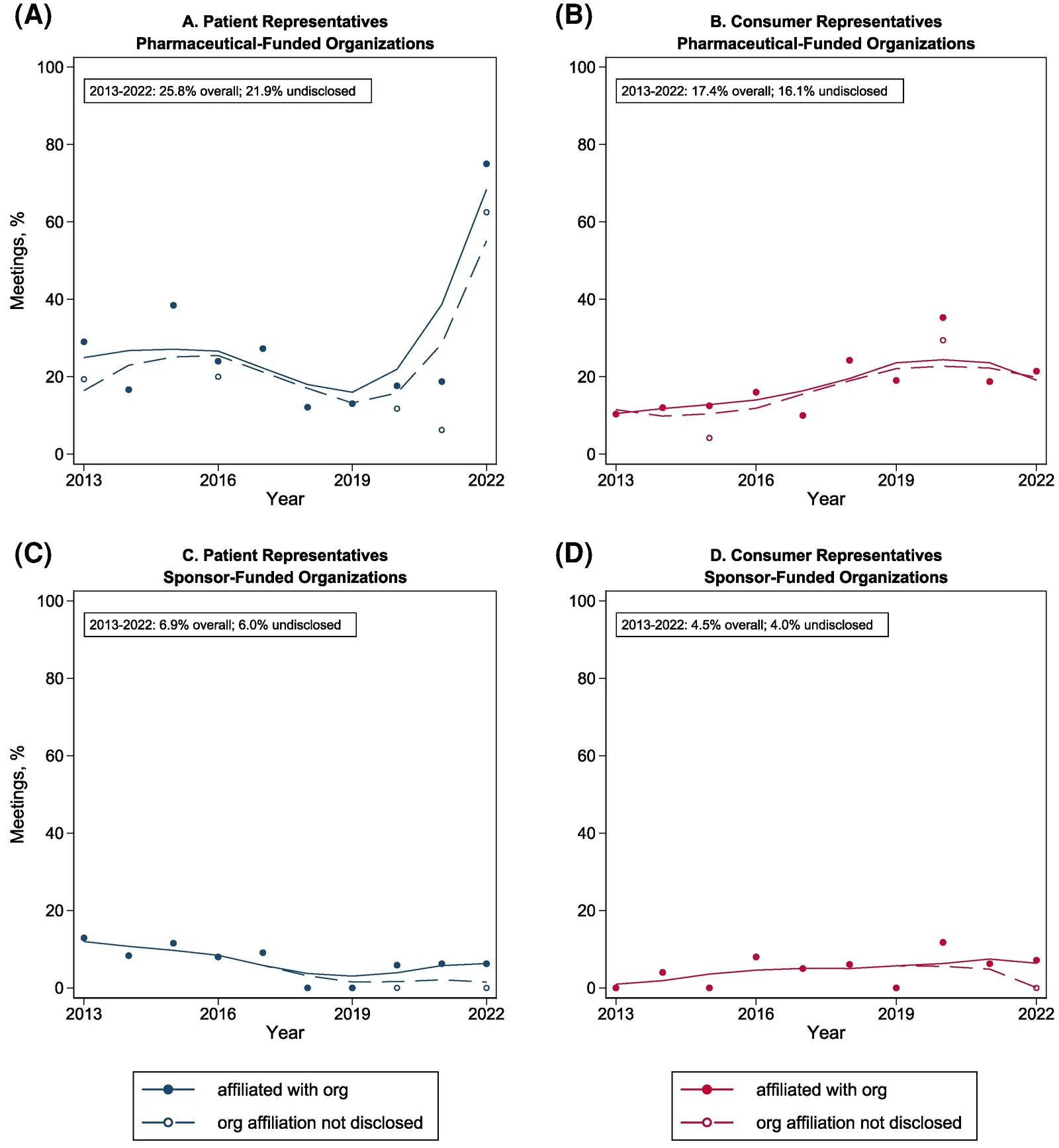
Share of meetings with representatives affiliated with advocacy organization receiving pharmaceutical or sponsor funding. Source: Authors’ analysis of FDA advisory committee meeting documents and original data collection on the organizational affiliations of patient and consumer representatives and the donors to those organizations, 2013-2022. Notes: Each solid dot represents the percentage of meetings in that year with at least one representative affiliated with an organization receiving pharmaceutical (or sponsor) funding. Each open dot represents the percentage of meetings in that year with at least one representative affiliated with an organization receiving pharmaceutical (or sponsor) funding and whose organizational affiliation was not disclosed at the meeting. Lines are lowess-smoothed trend lines. The top left corner of each panel reports average annual percentages for the full 2013-2022 period.

More than 1 in 10 (10.7%) meetings included either a patient or a consumer representative affiliated with an organization that received funding from the sponsor of the product under review (Appendix A3). About 7% of meetings included a patient representative, and 5% a consumer representative, affiliated with a sponsor-funded organization (Figure 1, Panels C and D).

All but one FDA advisory committee had meetings with patient representatives tied to pharmaceutical-funded advocacy organizations (Figure 2, Panel A). The committee with the greatest share of meetings involving patient representatives with such ties was the cardiovascular drugs committee (52.6%). Only the dermatologic/ophthalmic drugs committee had no meetings with patient representatives tied to pharmaceutical-funded organizations.

**Figure 2.**
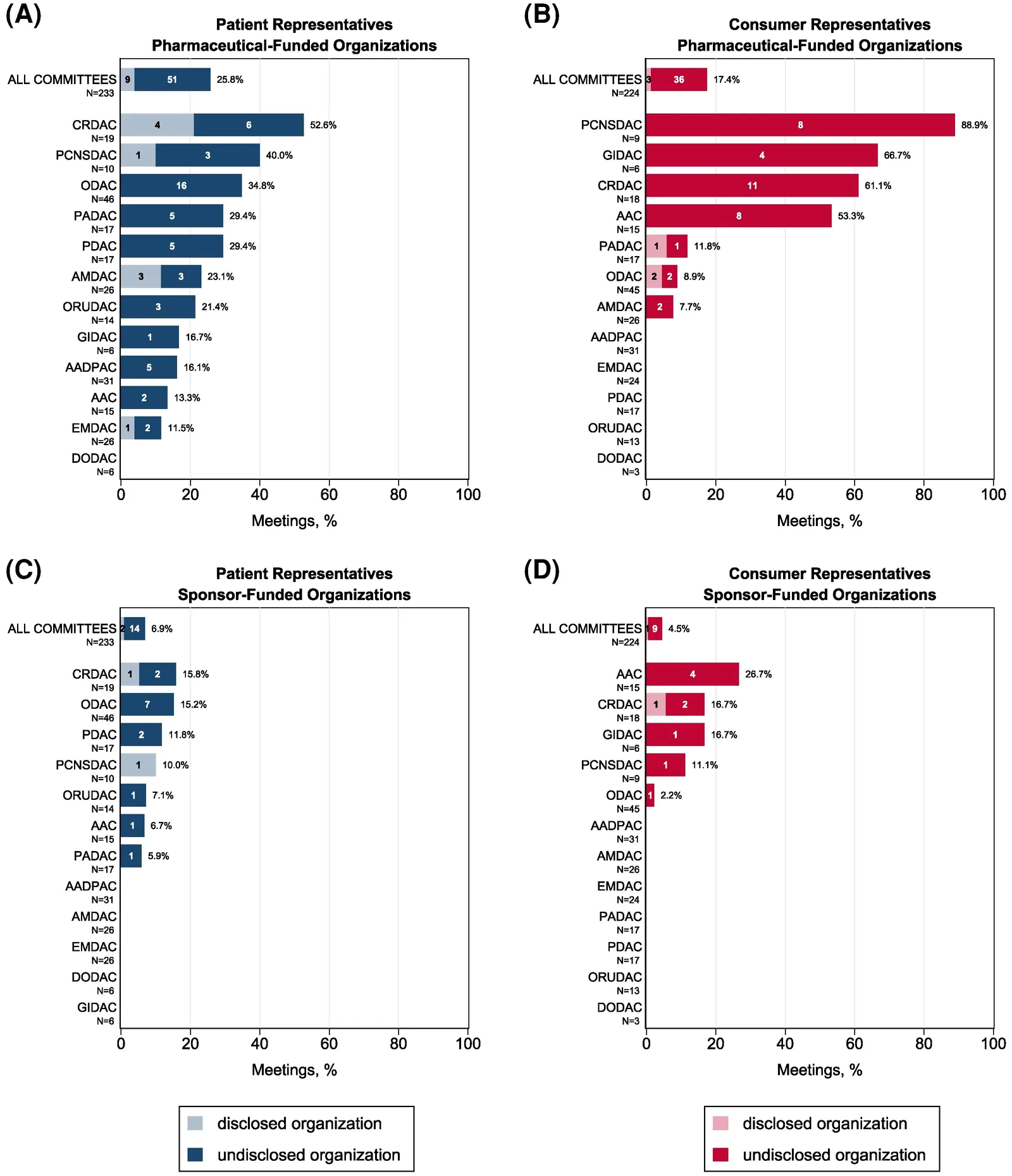
Share of meetings with representatives affiliated with advocacy organization receiving pharmaceutical or sponsor funding, by committee. Source: Authors’ analysis of FDA advisory committee meeting documents and original data collection on the organizational affiliations of patient and consumer representatives and the donors to those organizations, 2013-2022. Notes: Total length of each bar represents the percentage of meetings with at least one representative affiliated with an organization receiving pharmaceutical (or sponsor) funding. The percentage of meetings with at least one representative affiliated with an organization receiving pharmaceutical (or sponsor) funding is reported to the right of the bar. The light portion of the bar represents the percentage of meetings with at least one representative affiliated with an organization receiving pharmaceutical (or sponsor) funding and all such organizational affiliations were disclosed. The dark portion of the bar represents the percentage of meetings with at least one representative affiliated with an organization receiving pharmaceutical (or sponsor) funding and at least one such organizational affiliation went undisclosed at the meeting. The total number of meetings for each committee is reported under the committee's name on the *y*-axis. The number of meetings with disclosed and undisclosed affiliations with pharmaceutical (or sponsor) funded organizations is reported within each section of the bar. Committees are AAC (Arthritis Advisory Committee), AADPAC (Anesthetic and Analgesic Drug Products Advisory Committee), AMDAC (Antimicrobial Drugs Advisory Committee), CRDAC (Cardiovascular and Renal Drugs Advisory Committee), DODAC (Dermatologic and Ophthalmic Drugs Advisory Committee), EMDAC (Endocrinologic and Metabolic Drugs Advisory Committee), GIDAC (Gastrointestinal Drugs Advisory Committee), ODAC (Oncologic Drugs Advisory Committee), ORUDAC (Obstetrics, Reproductive and Urologic Drugs Advisory Committee), PADAC (Pulmonary-Allergy Drugs Advisory Committee), PCNSDAC (Peripheral and Central Nervous System Drugs Advisory Committee), and PDAC (Psychopharmacologic Drugs Advisory Committee).

Seven of 12 advisory committees had meetings with consumer representatives tied to pharmaceutical-funded advocacy organizations (Figure 2, Panel B). The committees with the greatest share of meetings involving consumer representatives with such ties were the nervous system drugs committee (88.9%) and the gastrointestinal drugs committee (66.7%).

There was similar variation across committees in the share of patient and consumer representatives affiliated with organizations receiving funding from drug sponsors (Figure 2, Panels C and D). For patient representatives, prevalence was highest in meetings of the cardiovascular drugs committee (15.8%) and the cancer drugs committee (15.2%). For consumer representatives, the highest share was in meetings of the arthritis drugs committee (26.7%).

## Discussion

We found that nearly 2 in 5 (38%) advisory committee meetings included either a patient or consumer representative affiliated with a pharmaceutical-funded advocacy organization. For several committees, this share was greater than 50%.

Prior research has found that 19% of patient and consumer representative appearances at FDA meetings between 2009 and 2012 had industry COIs of any kind, with 9% attributed to a relationship between industry and a patient advocacy organization.^9^ Our estimates of advocacy organization industry ties are considerably higher, perhaps due to our inclusion of a broader range of organizations and roles, as well as our use of a range of web sources to identify active involvement in these organizations.

At the same time, we found prevalence rates that are lower than the reported share (68%-83%) of patient advocacy groups receiving industry funding.^6,7^ These organizational-level funding rates, however, include support from device and biotechnology firms as well as pharmaceutical companies. In addition, the FDA could be making efforts to select patient and consumer representatives who are not connected to organizations known to receive industry funding.

We also examined patient and consumer representative affiliations to organizations that received funding from the drug sponsor in the year of the meeting. We found that more than 1 in 10 (11%) advisory committee meetings included a representative affiliated with a sponsor-funded advocacy organization. For several committees, this share was 15% or higher.

The ties to these sponsor-funded organizations were often not disclosed at advisory committee meetings. And even if representatives’ organizational affiliations were to be fully disclosed, there is currently no mechanism for the public to be informed of relevant organizational donors—even those posing a clear and direct risk of undue influence or bias, like the sponsor.

It is possible that, even if the public is not aware of these conflicts among patient and consumer representatives, the FDA is fully informed. In its 2016 draft guidance, the FDA highlights being an “active participant” in an organization that has a financial relationship with the sponsor as an “appearance issue.” This type of tie is not considered a conflict requiring recusal under Federal ethics laws but “may create the appearance that a member lacks impartiality.”^10^ Advisory committee members are required to disclose these relationships to the agency and may receive a Section 502 waiver permitting them to still participate. Unlike FDA's treatment of formal conflict-of-interest waivers, the FDA has never posted Section 502 waivers, which are issued at 27% of meetings.^11^ Thus, it is possible that some sponsor-funded organizational ties that we identified may have been permitted through the Section 502 waiver process.

It is also possible that at least some relevant organizational affiliations were not reported to the agency, since many affiliations were not publicly disclosed at meetings. Prior to each meeting, members are required to submit Form FDA 3410 with their financial disclosures. However, the section covering appearance issues could easily be overlooked, as it has a catch-all title of “Other Involvements,” occupies about 2% of the form, and does not request itemization. The instructions also do not explicitly mention organizational relationships, instead focusing on relationships more pertinent to scientific members such as “researcher initiated study” and “gift of research materials.”^12^

Pharmaceutical funding of and its influence on advocacy organizations are a growing public concern. FDA COI policies have not kept pace with these developments despite recent efforts to reform advisory committee processes. Finalizing guidance for relationships with advocacy organizations, revising Form FDA 3410 to more explicitly address both formal COIs and appearance issues related to these ties, and improving transparency through the public posting of Section 502 waivers would be important first steps in reinvigorating reform efforts.

A separate question is whether the organizational affiliations of patient and consumer representatives materially influence advisory committee proceedings and, further downstream, FDA actions. Even though patient and consumer representatives constitute only a small fraction of an advisory committee, votes from these representatives may play a pivotal role in close votes, and non-unanimous votes account for at least 60% of all votes.^13^ In addition, even though the agency does not always follow committee recommendations,^14,15^ and those recommendations are only one part of what the agency considers for any action, the FDA does pay close attention to the content of advisory committee discussions. According to the agency, “the main impact each member provides… is his or her contribution to the discussion.”^16^

This analysis was limited by the availability of online information that we were able to collect on the activities and affiliations of patient and consumer representatives as well as the availability and granularity of donor information reported by advocacy organizations. Both of these limitations in ascertainment would lead to prevalence underestimates. Because of data collection constraints, our analysis ends in 2022, so it may not reflect more recent practices. However, the prevalence rates we report were either stable or increasing over the preceding 10 years, so our reported rates are likely to represent lower bounds for more recent years.

## Conclusion

The organizational ties of patient and consumer representatives—and the financial relationships between their advocacy organizations and pharmaceutical companies—are an important but overlooked aspect of FDA advisory committees. The calculated prevalence rates show that these organizational relationships extend beyond isolated cases and signal a broader problem warranting FDA policy attention.

## Supplementary Material

qxag269_Supplementary_Data
