# Supplementary material for: Prevalence of ties to industry-funded organizations among patient and consumer representatives on FDA advisory committees
Source: Health Aff Sch. 2026 Oct 7;4(10):qxag269. doi: 10.1093/haschl/qxag269 (PMC13678205; doi:10.1093/haschl/qxag269)
Supplement: Supplementary file 1 [file qxag269_supplementary_data.zip › Industry_PAO_Prevalence_Appendix.pdf]

SUPPLEMENT TO:

Kanter GP, Mankowitz T. Prevalence of ties to industry-funded organizations among patient and consumer representatives in FDA advisory committees. *Health Aff Sch*. 2026.
https://doi.org/10.1093/haschl/qxag269

Appendix A1. Methodology

Appendix A2. Number and Percentage of Individuals With Organizational Affiliations

Appendix A3. Meeting-Level Prevalence of Organizational Affiliations

Appendix References

Appendix A1. Methodology

Patient and consumer representatives at FDA advisory committee meetings

We restricted our sample to CDER drug advisory committees with responsibility for key therapeutic classes. These committees included the Anesthetic and Analgesic Drug Products Advisory Committee (AADPAC), Antimicrobial Drugs Advisory Committee (AMDAC), Arthritis Advisory Committee (AAC), Cardiovascular and Renal Drugs Advisory Committee (CRDAC), Dermatologic and Ophthalmic Drugs Advisory Committee (DODAC), Endocrinologic and Metabolic Drugs Advisory Committee (EMDAC), Gastrointestinal Drugs Advisory Committee (GIDAC), Oncologic Drugs Advisory Committee (ODAC), Obstetrics, Reproductive and Urologic Drugs Advisory Committee (ORUDAC), Peripheral and Central Nervous System Drugs Advisory Committee (PCNSDAC), Psychopharmacologic Drugs Advisory Committee (PDAC), and the Pulmonary-Allergy Drugs Advisory Committee (PADAC). For these committees, we downloaded the meeting agendas, transcripts, rosters, and minutes from the FDA website for meetings held between 2013 and 2022.[1]

We defined distinct meetings based on the discrete topics listed on the meeting agenda, and identified voting meetings based on whether there was at least one vote held at the meeting as found in the transcript. For each meeting, we extracted the sponsor(s) of the product(s) discussed at the meeting from the meeting agenda or transcript. We extracted the organizational affiliations of patient and consumer representatives from the meeting roster and, using the meeting transcript, included any additional affiliations an attendee disclosed at the meeting.

Organizational affiliations of patient and consumer representatives

Other organizational affiliations of patient and consumer representatives – i.e., organizational affiliations not disclosed at FDA meetings – were obtained through online searches. Affiliations were identified using LinkedIn, Facebook, online articles, or organizational websites. The representative’s city and/or state listed on the meeting roster was used to identify the member’s LinkedIn or Facebook profile. Additional Google search terms included "FDA," "patient representative," or “consumer representative,” as well as the disease for which the drug under discussion was indicated and general terms related to the committee's therapeutic area (e.g., "cardiovascular" or "heart" for a CRDAC meeting).

Organizations were included only if they were patient advocacy organizations, patient support groups, patient informational groups, or medical research foundations. We identified these organizations by reviewing the description of activities on the organization’s website. Those describing their work as advocacy, support, education, or research were included.

Dates of affiliation were extracted from the representative’s LinkedIn profile or when explicitly mentioned in another source. Conflicting dates were resolved conservatively unless another source verified otherwise, and affiliations marked as current were considered as such unless an end date was independently confirmed. When exact dates of affiliation could not be determined, dates were imputed using the date of the representative’s curriculum vitae or the first and last archived webpage snapshots referencing the affiliation.

Affiliations were only included if: (1) the representative held a senior leadership role in the organization, such as board member, executive director, or founder (“executive leadership”); (2) their stated position title indicated leadership responsibilities (“other leadership”); or (3) there was evidence of repeated engagement and activities (“active involvement”). We included affiliations even if the roles appeared to be unpaid but excluded affiliations that were determined to be short-term in duration (i.e., less than 3 months), such as participation in annual events, awareness campaigns, training programs, and competitions. Donating to an organization was not considered to be an affiliation.

Affiliations were included if they were active in the calendar year of the representative’s meeting appearance.

Donors to patient and consumer advocacy organizations

For each organization and year, general donor data were initially extracted from a single source, either an annual report or organizational webpage. When donors were reported in both, source selection followed a defined hierarchy. Dated donor lists, whether from an annual report, webpage, or newsletter, were preferred over undated donor lists. As a result, annual reports were generally preferred over webpage snapshots. However, webpage snapshots were used instead when they included a more comprehensive list of donors or to remain consistent with calendar versus fiscal year donor reporting over time. Sources reporting dollar ranges were preferred over name ranges or undisclosed amounts, and annual donations were preferred over cumulative donations. If an organization reported both exact dollar donations from pharmaceutical companies and separately reported general donations within its annual report, both sources were extracted to ensure a comprehensive list of donors. Donors for specific events or programs were not extracted unless the organization mainly reported their donors by specific events and/or programs.

Donations reported in annual reports were assigned to the calendar year covering the majority of the report's donation period. For example, a donation in a fiscal year running 4/1/2019–3/31/2020 was assigned to calendar year 2019. A fiscal year beginning July 1 was treated as the same calendar year, so fiscal year 2019 was treated as calendar year 2019. Donations reported on the organization's website were assigned to the year of the page's last-updated date, or, if none, then the date of the archived snapshot. A contribution was counted regardless of dollar amount, since organizations frequently reported donations using dollar ranges or named tiers rather than exact figures.

Organizational donors were identified as pharmaceutical firms in a two-stage process. In the first stage, donor names were matched against a list of pharmaceutical and biotechnology firms selected using their North American Industry Classification System (NAICS) codes as found in the Gale Business Insights (Gale BI) database. The relevant NAICS categories included medicinal and botanical, pharmaceutical preparation, biological-product manufacturing, and biotechnology and life-sciences research and development. A donor was tagged as a pharmaceutical candidate if it matched this list via an exact name match or via a fuzzy name match. Additional donors were tagged as pharmaceutical candidates via an exact match of company partnerships (e.g., AstraZeneca Bristol Myers Squibb) or a pharmaceutical and biotechnology keyword match (e.g., "pharma," "therapeutics," "biopharma," "laboratories").

In the second stage, each of the 686 donors tagged as pharmaceutical candidates was manually reviewed. We excluded names of non-pharmaceutical manufacturers, including medical device and

vaccine manufacturers, other health product manufacturers, clinical laboratories, and dietary-supplement makers. We also excluded names used by both pharmaceutical and non-pharmaceutical firms that could not be disambiguated based on contextual information. We included names indicating corporate foundation giving but excluded corporate employee-giving programs. The remaining 509 donor names confirmed to be pharmaceutical manufacturers or their foundations, as well as the names of meeting sponsors, were then standardized to a single company name and linked to – when applicable – the names of their parent companies in each year between 2013 and 2022.

Pharmaceutical-funded and sponsor-funded organizational affiliations

Pharmaceutical donors to advocacy organizations were included if the representative's affiliation was active in the year of the meeting and the donation was made that same year (i.e., meeting year = affiliation year = donation year). If donors to an advocacy organization could not be identified for a given year, we assumed that the organization had no pharmaceutical company donors in that year. Donations from meeting sponsors were counted when the sponsor or its parent company matched to a donor or its parent company (i.e., any of the four sponsor–donor or parent-company combinations). Because a representative could appear at more than one meeting, affiliations and donations were evaluated separately for each meeting appearance.

Analytic sample

Of 236 distinct voting meetings, 234 comprised the analytic sample because 2 meetings included no patient representative and no consumer representative. The consumer representative from the Ophthalmic Devices Panel of the Medical Devices Advisory Committee (MDAC) at a joint meeting with DODAC was excluded because consumer representatives on MDAC panels do not have voting privileges. The meeting itself was retained in the sample because it included a patient representative.

One individual appeared as both a consumer representative and a patient representative. This individual served one term as a consumer representative, attending multiple meetings, and later appeared at a single meeting as a patient representative. They were included in meeting-level calculations in their specific consumer or patient representative role but classified as a consumer representative (their primary role) in person-level calculations with only their appearances as a consumer representative counted.

Appendix A2. Number and Percentage of Individuals With Organizational Affiliations

	Total	Patient	Consumer
No. representatives (N)	136	97	39
Affiliated with any advocacy org, n (%)			
Total	76 (55.9%)	57 (58.8%)	19 (48.7%)
Disclosed	13 (17.1%)	6 (10.5%)	7 (36.8%)
Undisclosed	63 (82.9%)	51 (89.5%)	12 (63.2%)
Executive leadership	43 (31.6%)	29 (29.9%)	14 (35.9%)
Disclosed	16 (37.2%)	7 (24.1%)	9 (64.3%)
Undisclosed	27 (62.8%)	22 (75.9%)	5 (35.7%)
Other leadership	26 (19.1%)	19 (19.6%)	7 (17.9%)
Disclosed	0 (0.0%)	0 (0.0%)	0 (0.0%)
Undisclosed	26 (100.0%)	19 (100.0%)	7 (100.0%)
Active involvement	25 (18.4%)	20 (20.6%)	5 (12.8%)
Disclosed	3 (12.0%)	2 (10.0%)	1 (20.0%)
Undisclosed	22 (88.0%)	18 (90.0%)	4 (80.0%)
Affiliated with advocacy org receiving pharmaceutical funding, n (%)			
Total	39 (28.7%)	31 (32.0%)	8 (20.5%)
Disclosed	4 (10.3%)	3 (9.7%)	1 (12.5%)
Undisclosed	35 (89.7%)	28 (90.3%)	7 (87.5%)
Executive leadership	6 (4.4%)	4 (4.1%)	2 (5.1%)
Disclosed	3 (50.0%)	2 (50.0%)	1 (50.0%)
Undisclosed	3 (50.0%)	2 (50.0%)	1 (50.0%)
Other leadership	16 (11.8%)	11 (11.3%)	5 (12.8%)
Disclosed	1 (6.2%)	0 (0.0%)	1 (20.0%)
Undisclosed	15 (93.8%)	11 (100.0%)	4 (80.0%)
Active involvement	19 (14.0%)	17 (17.5%)	2 (5.1%)
Disclosed	1 (5.3%)	1 (5.9%)	0 (0.0%)
Undisclosed	18 (94.7%)	16 (94.1%)	2 (100.0%)
Affiliated with advocacy org receiving sponsor funding, n (%)			
Total	20 (14.7%)	14 (14.4%)	6 (15.4%)
Disclosed	1 (5.0%)	1 (7.1%)	0 (0.0%)
Undisclosed	19 (95.0%)	13 (92.9%)	6 (100.0%)
Executive leadership	3 (2.2%)	2 (2.1%)	1 (2.6%)
Disclosed	1 (33.3%)	1 (50.0%)	0 (0.0%)
Undisclosed	2 (66.7%)	1 (50.0%)	1 (100.0%)
Other leadership	8 (5.9%)	4 (4.1%)	4 (10.3%)
Disclosed	1 (12.5%)	0 (0.0%)	1 (25.0%)
Undisclosed	7 (87.5%)	4 (100.0%)	3 (75.0%)
Active involvement	10 (7.4%)	8 (8.2%)	2 (5.1%)
Disclosed	0 (0.0%)	0 (0.0%)	0 (0.0%)
Undisclosed	10 (100.0%)	8 (100.0%)	2 (100.0%)

Source: Authors' analysis of FDA advisory committee meeting documents and original data collection on the organizational affiliations of patient and consumer representatives and the donors to those organizations, 2013-2022. **Notes**: This table

reports the number and percentage of representatives with (1) at least one affiliation in effect in the year of their meeting appearance; (2) at least one affiliation with an organization receiving pharma funding; and (3) at least one affiliation with an organization receiving sponsor funding. Within each of these three groups, the table also reports the number and share of representatives by level of affiliation. Affiliations were considered to be “executive leadership” if the representative held a senior leadership role in the organization, such as board member, executive director, or founder; “other leadership” if their stated position title indicated leadership responsibilities; and “active involvement” if there was evidence of repeated engagement and activities. A representative is counted at every level at which they held a qualifying affiliation, so the level rows are not mutually exclusive and may sum to more than the group Total. Percentages in the Total row and level rows are calculated based on all representatives in the column. Disclosed rows report the number of representatives whose affiliations in that row’s category were always disclosed at meetings. Undisclosed rows report the number of representatives who had at least one affiliation in that row’s category which went undisclosed at a meeting. Percentages in the Disclosed and Undisclosed rows are calculated based on the number of representatives in the Total or level row directly above, so each Disclosed/Undisclosed pair sums to 100%. Disclosure is assessed separately for each funding group and each level, so a representative may be counted as Disclosed at one level and Undisclosed at another level. One individual, who served as a consumer representative in most meetings and as a patient representative in a different meeting, is classified as a consumer representative, and only their appearances as a consumer representative are counted in this person-level table.

Appendix A3. Meeting-Level Prevalence of Organizational Affiliations

	Patient or Consumer	Patient	Consumer
No. meetings (N)	234	233	224
Affiliated with any advocacy org, n (%)			
Total (Disclosed + Undisclosed)	186 (79.5%)	140 (60.1%)	110 (49.1%)
Undisclosed	149 (63.7%)	111 (47.6%)	67 (29.9%)
Executive leadership	137 (58.5%)	85 (36.5%)	81 (36.2%)
Undisclosed	79 (33.8%)	59 (25.3%)	29 (12.9%)
Other leadership	74 (31.6%)	46 (19.7%)	35 (15.6%)
Undisclosed	70 (29.9%)	41 (17.6%)	34 (15.2%)
Active involvement	70 (29.9%)	40 (17.2%)	34 (15.2%)
Undisclosed	60 (25.6%)	35 (15.0%)	26 (11.6%)
Affiliated with advocacy org receiving pharmaceutical funding, n (%)			
Total (Disclosed + Undisclosed)	89 (38.0%)	60 (25.8%)	39 (17.4%)
Undisclosed	83 (35.5%)	51 (21.9%)	36 (16.1%)
Executive leadership	17 (7.3%)	10 (4.3%)	7 (3.1%)
Undisclosed	8 (3.4%)	3 (1.3%)	5 (2.2%)
Other leadership	44 (18.8%)	22 (9.4%)	23 (10.3%)
Undisclosed	35 (15.0%)	22 (9.4%)	14 (6.2%)
Active involvement	46 (19.7%)	29 (12.4%)	17 (7.6%)
Undisclosed	44 (18.8%)	27 (11.6%)	17 (7.6%)
Affiliated with advocacy org receiving sponsor funding, n (%)			
Total (Disclosed + Undisclosed)	25 (10.7%)	16 (6.9%)	10 (4.5%)
Undisclosed	23 (9.8%)	14 (6.0%)	9 (4.0%)
Executive leadership	3 (1.3%)	2 (0.9%)	1 (0.4%)
Undisclosed	2 (0.9%)	1 (0.4%)	1 (0.4%)
Other leadership	13 (5.6%)	5 (2.1%)	8 (3.6%)
Undisclosed	11 (4.7%)	5 (2.1%)	6 (2.7%)
Active involvement	11 (4.7%)	9 (3.9%)	2 (0.9%)
Undisclosed	10 (4.3%)	8 (3.4%)	2 (0.9%)

Source: Authors' analysis of FDA advisory committee meeting documents and original data collection on the organizational affiliations of patient and consumer representatives and the donors to those organizations, 2013-2022. **Notes**: This table reports the number and percentage of meetings with (1) at least one representative with an affiliation in effect in the year of the meeting; (2) at least one representative affiliated with an organization receiving pharma funding; and (3) at least one representative affiliated with an organization receiving sponsor funding. Within each of these three groups, the table also reports the number and share of meetings by level of affiliation. Affiliations were considered to be "executive leadership" if the representative held a senior leadership role in the organization, such as board member, executive director, or founder; "other leadership" if their stated position title indicated leadership responsibilities; and "active involvement" if there was evidence of repeated engagement and activities. A meeting is counted at every level at which the attending representative(s) held a qualifying affiliation, so the level rows are not mutually exclusive and may sum to more than the group Total. Undisclosed rows report the number and percentage of meetings in that row's category with at least one attending representative who did not disclose an affiliation. Disclosure is assessed separately for each funding group and each level, so a meeting may be counted as Undisclosed in one row and not another. All percentages are calculated based on all meetings in the column.

APPENDIX REFERENCES

1. US Food and Drug Administration. Human drug advisory committees. Updated October 22, 2024. Accessed September 25, 2026. https://www.fda.gov/advisory-committees/committees-and-meeting-materials/human-drug-advisory-committees
